# A Glimpse into Dendrimers Integration in Cancer Imaging and Theranostics

**DOI:** 10.3390/ijms24065430

**Published:** 2023-03-12

**Authors:** Adriana Cruz, José Barbosa, Patrícia Antunes, Vasco D. B. Bonifácio, Sandra N. Pinto

**Affiliations:** 1 iBB-Institute for Bioengineering and Biosciences and i4HB-Institute for Health and Bioeconomy, Instituto Superior Técnico, Universidade de Lisboa, Av. Rovisco Pais, 1049-001 Lisboa, Portugal; 2 Bioengineering Department, Instituto Superior Técnico, Universidade de Lisboa, Av. Rovisco Pais, 1049-001 Lisboa, Portugal

**Keywords:** cancer, nanocarriers, anticancer dendrimers, intracellular targeting, theranostics

## Abstract

Cancer is a result of abnormal cell proliferation. This pathology is a serious health problem since it is a leading cause of death worldwide. Current anti-cancer therapies rely on surgery, radiation, and chemotherapy. However, these treatments still present major associated problems, namely the absence of specificity. Thus, it is urgent to develop novel therapeutic strategies. Nanoparticles, particularly dendrimers, have been paving their way to the front line of cancer treatment, mostly for drug and gene delivery, diagnosis, and disease monitoring. This is mainly derived from their high versatility, which results from their ability to undergo distinct surface functionalization, leading to improved performance. In recent years, the anticancer and antimetastatic capacities of dendrimers have been discovered, opening new frontiers to dendrimer-based chemotherapeutics. In the present review, we summarize the intrinsic anticancer activity of different dendrimers as well as their use as nanocarriers in cancer diagnostics and treatment.

## 1. Introduction

All types of human cells may suffer an abnormal proliferation that can lead to cancer cells. Cancer classification/identification is performed according to the tissue and cell type from which the cancer cells arise. Therefore, there are multiple distinct types of cancer, which can vary significantly in their behavior and response to treatments [1]. This disease is a major public health problem and a leading cause of death worldwide in countries of all income levels. 

According to the World Health Organization (WHO), the social and economic impact caused by cancer is increasing. In a 2014 report, an annual cost of EUR 1.04 trillion was estimated for global cancer expenses. The report also declared that it is important to continue investing in care and control, which will prevent a considerable number of deaths for years to come. [2]. Extended lifespan associated with environmental factors (e.g., exposure to pollution, carcinogenic agents, radiation, viruses and bacteria) and the low efficacy of available treatments, closely associated with an increased drug resistance, also contributes to cancer development [3]. 

Currently, the standard cancer treatments used in clinical settings are radiotherapy, surgery, and conventional chemotherapy [4]. However, these therapies present several drawbacks, such as high toxicity, due to insufficient selectivity and unspecific targeting of cancer cells, which leads to increased resistance to anticancer drugs.

It is therefore relevant to find new anticancer agents able to control tumor growth with minimal side effects. In recent years, the use of nanotechnology in cancer treatment has offered some exciting possibilities, including improvement of detection and elimination of cancer cells before tumor development. This includes the use of dendrimer-based nanotherapeutics as a novel strategy for diagnosis and therapy (theranostics) [5,6,7]. Dendrimers are synthetic 3D polymers with well-defined layered architecture [8]. Owing to their high functionality and loading capacity, as well as their precisely controlled chemical composition and molecular weight, dendrimer-based anticancer therapies offer great advantages over conventional formulations. These include the use of dendrimers as advanced contrast agents (diagnosis) [6], nanocarriers (treatment) [5,9], and theranostic agents (diagnosis, treatment and disease monitoring) [7]. In the quest for new anticancer drugs using dendrimer-based therapies, Shao et al. demonstrated that dendrimers may display innate anticancer activity and anti-metastatic properties without the loading of any therapeutic agent [10]. In this study, poly(acylthiourea) G4 dendrimer (PATU-PEG) demonstrated a higher anticancer activity than Doxil^®^ (doxorubicin liposomal), a well-established chemotherapy drug, and was able to inhibit cell seeding, thus exhibiting a strong decrease in cell metastasis. This study opened new doors for anticancer dendrimer design, targeting effective and safer (with reduced toxicity) anticancer therapies that may be combined with diagnostic features, such as novel molecular imaging technologies. In summary, due to the increase relevance of dendrimers in therapy, this review highlights recent studies regarding the use of these outstanding nanoparticles in cancer theranostics. We also discuss the intracellular pathways associated with their cellular uptake and possible organelle targeting.

## 2. Dendrimer Nanoparticles 

**Figure 1:**
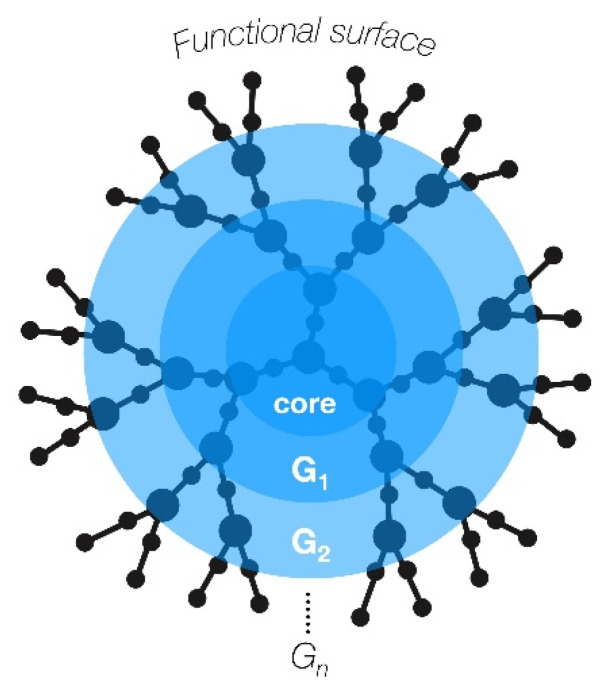
Schematic representation of a dendrimer nanoparticle, showing the core, repetitive branching units (dendrons) that constitute the growing layers (generations, G) and terminal groups (functional surface).

Multifunctional nanoparticles display great potential for drug and gene delivery, especially for cancer therapy [11,12]. Dendrimers are a class of hyperbranched synthetic polymers with a very low polydispersity, also known as “cascade polymers” [13]. Biologically, dendrimers are highly biocompatible, with a predictable biodistribution and cell-membrane-interacting features mostly determined by their size and surface charge [14]. They were first synthesized in the late 1970s by Tomalia et al., with the desire to mimic a common pattern in nature with vast potential applications [15]. Due to their hyperbranched structure, dendrimers are extremely versatile macromolecules. Their structure can be defined by three main elements: the inner core, repetitive branching units (dendrons), and terminal groups that provide surface tuning (Figure 1) [16].

Dendrimers are classically obtained by two main approaches: a divergent or a convergent synthesis. In the first methodology, the dendrimer structure is constructed starting from a core molecule. The core reacts with monomers containing one reactive group and two dormant groups originating the first generation (G1) dendrimer. Then, the periphery of the G1 dendrimer may be activated for reaction with more monomers forming the second-generation dendrimer (G2), and so on. The divergent approach typically originates lower dendrimer generations of open structures and asymmetric shape [16]. In the convergent approach, the individual branches (dendrons) are first synthetized and then attached to a functional core molecule [17]. This methodology minimizes the occurrence of structural problems and facilitates the purification of the product [16]. Later, with the development of “click chemistry” [18], the preparation of dendronized systems became more efficient, requiring minimal purification [19]. She et al. [20], for instance, synthetized G2-poly-L-lysine dendrons that were connected at the core to a heparin sulphate moiety via “click chemistry”, and doxorubicin (DOX) was conjugated to the terminal ends using acyl hydrazine. As a consequence of the dendrimer architecture, the number of peripheral groups increases exponentially with generations, which results in nanosized particles suitable for drug loading and release [21]. However, when a critical branched state is reached, dendrimers cannot grow further because of the steric restriction imposed by the increasing branch density. This phenomenon is known as the “starburst effect” and is usually observed in high generations [16].

Since the discovery of these polymers [15], a variety of dendrimers have been developed, polyamidoamine (PAMAM) being the most studied [22]. PAMAM dendrimers are synthetized by the divergent method, mostly using an ethylenediamine core, and are hydrophilic, biocompatible, and non-immunogenic. Polypropylene imine (PPI) dendrimers, along with PAMAM, have been also widely investigated. PPIs are based on a 1,4-diaminobutane core or similar molecules and grow via double Michael addition reactions [13]. Poly-L-lysine (PLL) dendrimers are amino-acid-based polymers [23] that differ from PAMAM and PPI dendrimers in shape, since they are mostly asymmetrical. They have lysines as branching units and amines as terminal groups [22]. Phosphorous-based dendrimers are another interesting and well-studied class of dendrimers [24]. The potential of phosphorous dendrimers has been largely demonstrated, especially as cancer therapeutics. Similarly, carbosilane dendrimers have been explored as antimetastatic agents when complexed with ruthenium derivatives [25]. 

The different classes of dendrimers and their chemical structures are summarized in Table 1.

## 3. Dendrimers General Role in Cancer

Dendrimers can be used in a vast number of theranostic applications [26,27,28,29]. It is, therefore, important to be aware of the properties they have to display in order to be employed as biomedical devices. Biocompatibility is crucial to preventing undesirable responses from the host, a property that can only be defined depending on specific applications [29]. In order to prevent bioaccumulation and consequent toxicity, biodegradability is a must. Another important aspect for the development of biomedical devices is their pharmacokinetics, namely their fate in the body after administration [29]. Additionally, the water solubility of dendrimer–drug conjugates enhance the bioavailability of poorly soluble drugs [30]. Lastly, polyvalence, i.e., the ability to support versatile surface functionalization and multiple interactions with biological receptors, is a key property for highly versatile platforms [27].

Dendrimers, as shown in several studies, have a high potential to be used as nanocarriers for both diagnostic and therapeutic approaches [31,32]. Dendrimer–drug interactions might occur in many ways and are dependent on multiple factors such as size, charge, or the chemical nature of the dendrimer/drug. The chemistry behind nanocarriers is the same used in diagnostic or therapeutic schemes, with the agent selected for conjugation being the key player. In general, dendrimers could be used as nanocarriers via two major approaches: loading or conjugation at the surface of the drug and/or target molecule. Encapsulation solves solubility problems indicated by many chemotherapeutics and drugs in general. When a drug is entrapped into the dendrimer’s cavity, the polymer works as a dendritic box [33,34]; in this case, the dendrimers can cargo the drug of interest by forming structures that are stabilized via non-covalent interactions. In a different context, dendrimers could also be used as gene vectors, especially cationic dendrimers. When the strategy is dendrimer–drug conjugation, systemic effects can be reduced, increasing the efficacy of cellular targeting. This strategy also improves the half-life of the drug. The conjugate linker is also key to understanding release mechanisms. In many cases, ester and amide conjugate linkers are used that allow enzymatic or hydrolytic cleavage [34,35], easier for esters than amides [36]. Dendrimer–drug conjugation may influence the efficacy of drug itself. Importantly, these nano-polymers can cross cellular barriers by transcellular or paracellular pathways.

To use dendrimers as an alternative diagnostic tool or improve the properties of a contrast agent, it is important to guarantee some criteria. Dendrimers offer many advantages to improve the free delivery of contrast agents or drugs, including high solubility and low polydispersity, which are properties of all dendrimer classes (Table 2).

### 3.1. Dendrimers Role in Bioimaging and Theranostics

Since late-stage diseases are highly lethal, early detection is vital for successful treatment. Hence, the role of dendrimers in diagnosis is of great value due to their versatility and polyvalence. The most common techniques in molecular imaging used for diagnostic purposes are computed tomography (CT), magnetic resonance imaging (MRI), and positron emission tomography (PET) using contrast agents (CAs). In addition, contrast agents can also be used in theranostic approaches via molecular imaging such as photodynamic therapy (PDT), photothermal therapy (PTT) or neutron capture therapy (NCT) [51,52,53]. MRI is non-invasive and provides high-quality three-dimensional images without causing radiation damage, thus, it is used to detect solid tumors. In some body tissues, the use of CAs is needed to enhance the intensity of the signal transmitted, improving the quality of the image obtained and the sensitivity of the method. The most used and studied CAs for cancer diagnosis are gadolinium (Gd) chelates. However, CAs have some limitations, such as poor solubility, a low penetration rate, and in some cases, high toxicity. In this sense, nanoparticles and dendrimers have been emerging as good alternatives to overcome these drawbacks. For instance, conjugation of Gd chelates to a dendrimer improved circulation time and specificity in comparison to free Gd chelates [28]. Wiener et al. developed a new class of MRI-CAs with enhanced magnetic resonance properties through the conjugation of a PAMAM_G6_ dendrimer with a Gd (III)- DTPA by means of thiourea linkage, in which DTPA was a chelator. The authors obtained excellent MRI images of blood vessels and long blood circulation times [6]. In addition, PLL dendrimers were also developed for MRI via conjugation with Gd-DTPA [54]. 

With the progression of nanomedicine, one major goal has been the development of nanodevices capable of simultaneously combining therapy and diagnosis, allowing real-time monitoring of treatment progress and efficacy. This theranostic approach enables personalized medicine directing the treatment for each patient. This theranostic paradigm implies the acknowledgement that not everyone responds in the same way to a certain drug or treatment protocol [55,56]. Nanoparticles have been proposed and demonstrated as potential nanovehicles for cancer theranostics. Several nanoparticles were developed and approved for this aim, but in some cases they are not enough to overcome pharmacokinetic limitations [57]. Dendrimers, in particular, are one of the most suitable and promising platforms under this strategy, since they can be designed to overcome many of these drawbacks. For diagnostic purposes, the major contribution of dendrimers is in molecular imaging, where they are used as MRI, PET, CT and luminescent imaging agents. PAMAM dendrimers are the most studied since they were the first to be synthetized and are commercially available. They are also used in PDT or PTT techniques as well as agents of NCT. 

For bioimaging and theranostics approaches, CAs are, in general, conjugated with dendrimers by the addition of a chelator agent. Dendrimers and dendrons can be conjugated with radionuclides such ^18^F for PET [32]. For breast cancer MRI, a Gd-DO3A agent was conjugated with a PAMAM_G6_-Cystamine dendrimer, enabling an increased concentrations of Gd-DO3A in the blood circulation [58]. For PTT applications targeting breast cancer, PAMAM_G3_ and PAMAM_G5_ were conjugated with Au nanorods@SiO_2_ and MoS_2_, respectively [59,60]. For human colorectal carcinoma, PPI dendrimers were reported as MRI contrast agents by conjugation with a DTPA derivative complexed with Gd(III) [60].

Dendrimers functionalized with chelating agents can be readily labeled with radioisotopes. For example, ^68^Ga was conjugated with a PAMAM_G4_-DOTA with a higher retention of the conjugate in Ehrlich’s ascites tumor models in pre-clinical trials [61]. Conjugation with fluorescent probes is also a reliable approach. Folic-acid-functionalized PAMAM_G5_ conjugated with fluorescein isothiocyanate (FITC) (FI-FA-PAMAM_G5_) were developed for targeting folate-receptor-overexpressing cancer cells. To make this nanoplatform suitable for PET, FI-FA-PAMAM_G5_ was conjugated with the chelator agent DOTA and ^64^Cu (^64^Cu-DOTA-FI-FA-PAMAM_G5_). The final multifunctional nanoplatform shows promising results regarding diagnosis of FR-overexpressing tumor xenografts [62]. Encapsulation with fluorescent probes is also useful for diagnostic purposes. Increasing specificity with molecular targets at the surface, probes are released from the dendritic box and delivered into the target tissue. For instance, FITC or rhodamine dyes were encapsulated in functionalized (lauroyl and propranolol) PAMAM dendrimers and used for detection of colon and breast cancers [63,64]. Other examples and techniques have been reviewed [39,65].

The conjugation strategy has also been widely explored. For breast cancer MRI, a Gd-DO3A agent was conjugated with a PAMAM_G6_-Cystamine dendrimer, enabling an increased concentration of Gd-DO3A in blood circulation [58]. For PDT or PTT applications targeting breast cancer, PAMAM_G3_ and PAMAM_G5_ were conjugated with MoS_2_ and Au nanorods@SiO_2_, respectively [59,60]. Dendrimers functionalized with chelating groups readily conjugate radioisotopes. For example, ^68^Ga was conjugated with a PAMAM_G4_-DOTA for breast cancer diagnosis, and this demonstrated a higher retention of the conjugate in the tumor tissue [61]. 

Table 3 summarizes the use of dendrimers for bioimaging and theranostic applications.

Although dendrimers have a key role in diagnostics, there are still some concerns regarding their safety. The main issues are biodistribution and elimination, but toxicity may be relevant in the case of intrinsically cationic dendrimers, as they are dependent on size/generation/M_w_/charge as shown by us [49] and others [50]. 

The biological fate of polymers is the sum of many factors and since there is still a gap of knowledge regarding how their physicochemical properties (size, charge, degree of branching, shape, plasma coating) influence their biological fate, radiolabeling is normally required at the different steps of synthesis, as well as extensive studies on different animal models. Nevertheless, for PAMAM dendrimers, a rapid decrease in blood plasma concentration with binding to vascular surfaces and accumulation in the liver, kidneys, and spleen was observed, as well as rapid opsonization and phagocytic clearance by the reticuloendothelial system [66,67]. There is evidence that some dendrimers can still be metabolized to their native constituents, but small alterations by capping or degree of branching can render them inert to biodegradation [68]. 

Also relevant is the fact that some surface modifications such as preparation of conjugates made with PEG allow for longer blood circulation and renal clearance and lower toxicity [39,69].

**Table 3:** The use of dendrimers, selected in this review, for bioimaging and theranostic applications.

Dendrimers	Technique	In Vitro Cell Cancer Model	Reference
^18^F-PAMAM dendrons	PET	Breast cancer (MDA-MB-435, MDA-MB-468 and SKBr3 cells)	[32]
PAMAM_G6_-Cystamine- (Gd-DO3A)	MRI	Breast cancer (MDA-MB-231 cells)	[58]
PAMAM_G5_-MoS_2_	PTT	Breast cancer (4T1 cells)	[59]
AuNRs@SiO_2_-PAMAM_G3_	PTT	Breast cancer (MCF-7 cells)	[60]
DAB-Am64-(1B4M-Gd)_64_	MRI	Colorectal carcinoma (LS174T cells)	[70]
^68^Ga-PAMAM_G4_-DOTA	PET	Undifferentiated tumor (Ehrlich’s ascites tumor cell lines)	[61]
^64^Cu-DOTA-FA-FI-PAMAM_G5_-NHAc	PET	Lung adenocarcinoma (KB and A549 cells)	[62]

### 3.2. Dendrimers as Drug Nanocarriers

The main objective of drug conjugation to a nanocarrier is to improve its efficiency by enhancing aqueous solubility; increase circulation time; stabilize the drug; confer drug release mechanisms; and enable targeted delivery to specific tissues, thus reducing side effects, possibly decreasing drug dosage, and aiding in the passage through biological barriers [26]. In addition, in cancer therapeutics it is very challenging to find adequate treatment that is highly specific for neoplastic tissues. Due to lack of specificity, anticancer drugs are administered systemically, with several side effects [34]. To overcome such problems, several nanoparticles have been used in chemotherapy, such as liposomes, nanotubes, or polymer-drug conjugates. Dendrimers such as PAMAM, PPI, PLL, and polyether have then been also investigated for the delivery of anticancer agents [71,72]. 

In this context, DOX-dendrimer conjugates were tested for the treatment of lung cancer. DOX-PAMAM_G4_ dendrimer was evaluated against a lung cancer metastasis model (B16-F10 melanoma cells). The results indicate that DOX-PAMAM_G4_ accumulated in lungs and the tumor burden decreased [73]. 

Controlled release in dendrimers is still a challenge, and the design of stimuli-responsive dendrimers is foreseen as a great solution [74]. For example, bortezomib (Btz) is a chemotherapeutic for the treatment of multiple myeloma; however, it was reported that administration of this drug can cause cardiotoxicity and thrombocytopenia [74]. Wang et al. [75] conjugated Btz with a catechol-functionalized PAMAM dendrimer via a boronate ester, which is an acid-labile group. The yielding prodrug was found to be stable at physiological pH and exhibited fast drug release due to tumor extracellular acidity. This “on-off” drug release system can improve therapeutic results, minimizing the side effects of the conventional drugs, possibly by drug conjugation (macromolecular complexes). 

Importantly, gemcitabine-loaded YIGSR-CMCht/PAMAM [(carboxymethylchitosan/poly(amidoamine) (CMCht/PAMAM) dendrimer nanoparticles functionalized with YIGSR laminin receptor binding peptide)] dendrimers induce high mortality in HCT-116 cancer cells in a co-culture model with L929 fibroblasts (healthy model cells), thus showing the high selectivity of this nanodrug delivery system [76]. 

Taking advantage of a multifunctional platform, some authors improved molecular targeting by adding extra ligands. For instance, PAMAM dendrimers were conjugated with the antibody trastuzumab (TZ) and DOX (DOX-TZ-PAMAM) to improve specificity against breast cancer cells. The half-maximal inhibitory concentration (IC_50_) was lower for DOZ-TZ-PAMAM than DOX-PAMAM, indicating the advantages of this strategy [77]. In another interesting approach, paclitaxel (PTX)-biotinylated PAMAM complexes were tested against ovarian cancer cells OVCAR-3 and human embryonic kidney cells HEK293T. The results revealed an increased uptake by cancer cells and a decrease in cytotoxicity for the biotinylated complexes [78]. 

Quintana et al. used a functionalized dendrimer to target the KB cell line. The nanodevice was composed of a generation 5 PAMAM dendrimer conjugated with folic acid (FA), FITC, and methotrexate (MTX), a chemotherapy agent and immune system suppressant (FA-FTIC-MTX-PAMAM_G5_). The human epithelial carcinoma KB cell line is known to overexpress folate receptors. The results of internalization and KB cell survival indicated an enhanced efficacy for the nanodevice when compared to free MTX [5].

PPI dendrimers are also reported as good nanocarriers for anticancer drugs [79]. These dendrimers are hemolytic, but their toxicity was found to be reduced by FA conjugation. Recently, a complex system of a PPI_G5_ conjugated with FA and MTX (FA-MTX-PPI _G5_) was developed. The data show a enhanced internalization and higher cytotoxicity towards the MCF-7 breast cancer cell line [80]. PPI dendrimers were also complexed with the antibody mAbK1 and PTX, a system that allowed a significant reduction in cancer activity and displayed a higher therapeutic index [81].

PPL dendrimers, being amphiphilic macromolecules, are often used as gene carriers [82] but since terminal lysines are easily modified, they can also work as nano-vehicles for anticancer drugs. PEGylated PLL containing docetaxel (DTX) is in Phase I clinical trials, being effective against different types of tumors [31]. Cationic PLL dendrimers are also useful for the encapsulation of other anticancer drugs such as 5-fluorouracil [83].

**Figure 2:**
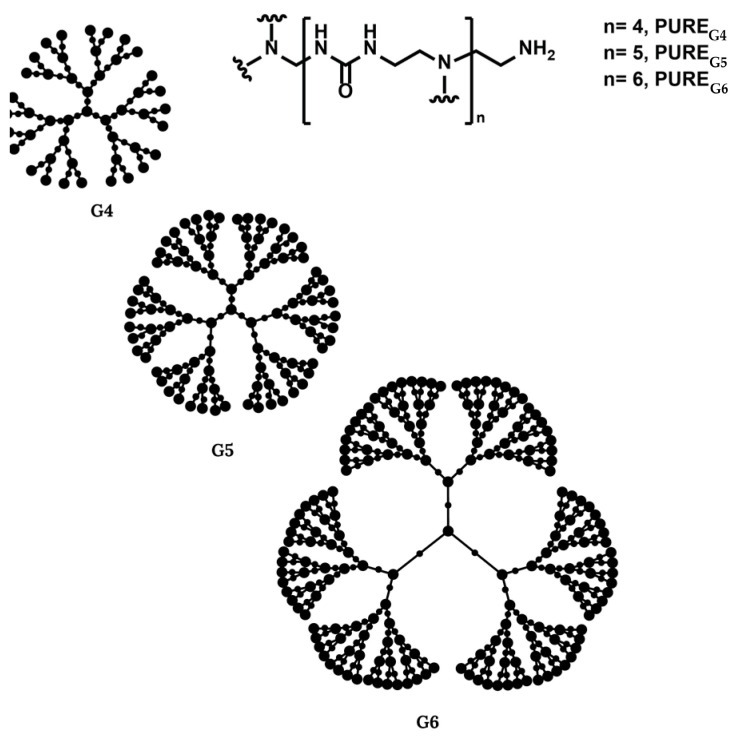
Schematic representation of different polyurea (PURE) dendrimer generations used as drug and gene delivery nanocarriers.

Polyurea (PURE) dendrimers (Figure 2), developed by our group, are another class of dendrimers with great potential for cancer therapeutics. PURE_G4_-OMeOx_48_ and PURE_G4_-OEtOx_48_ dendrimers, surface-coated with oligo-oxazoline PEG substitutes, were used as paclitaxel (PTX) nanocarriers. Free PTX had a IC_50_ of 0.094 µM after 48 h of incubation against the hepatocellular carcinoma cell line HepG2, a value that was dramatically reduced to 1.23 nM and 1.90 nM after encapsulation into PURE_G4_-OEtOx_48_ and PURE_G4_-OMeOx_48_, respectively [84]. The encapsulation of other anticancer agents into PURE dendrimers was also successfully achieved, as in the case of the nano-in-microparticles developed for inhalation chemotherapy [85].

### 3.3. Dendrimers as Gene Nanocarriers

Therapeutic nucleic acids are designed to trigger or suppress the expression of specific genes that are responsible for the biosynthesis of different proteins [86]. Nucleic acid therapy for cancer treatment has a lot of potential since nucleic acids are highly biocompatible and have high specificity compared to chemotherapies. However, nucleic acids are large hydrophilic molecules that cannot penetrate cell membranes and are vulnerable to enzymatic degradation; therefore, delivery systems are necessary to obtain good therapeutic outcomes [22]. 

Several distinctive properties of dendrimers make them a better option than other cationic polymers: a high density of positive charges provides multiple attaching sites for (negatively charged) nucleic acid molecules; the complexation of nucleic acids with dendrimers protects them from nuclease degradation [22]; and the abundance of tertiary amines facilitates endosomal disruption though a “proton sponge” effect, with consequent nucleic acids release [22,87].

Xu et al. [88] constructed a targeted delivery system by conjugating FA onto a PAMAM_G4_ surface to function as a DNA plasmid gene carrier for gene delivery into head and neck cancer cells. FA receptors are overexpressed in cancer cells, and because of that, FA is often used to target these cells [5]. The PAMAM_G4_-FA/plasmid polyplexes were taken up by receptor-mediated endocytosis. The authors verified that the conjugate exhibited a high tumor uptake and a highly localized retention in the tumors. By associating an anticancer drug with oligonucleotides, a synergistic effect was obtained [22]. Han et al. [89] combined drug and gene delivery by using a PAMAM dendrimer with encapsulated DOX associated with small interfering RNAs (siRNAs) targeting major vault protein (MVP), which is related to multidrug resistance. siRNAs suppress expression of carcinogenic genes by targeting messenger RNA (mRNA) expression [82]. PAMAM dendrimers were functionalized by a polysaccharide hyaluronic acid (HA) to increase tumor specificity since HA receptors are overexpressed in some types of cancer. For instance, it was observed that codelivery of siRNA and DOX by PAMAM-HA caused an increase in cytotoxicity in MCF-7 cells, since the delivery of siRNA allowed DOX to access the cell nucleus more easily [89].

Our group also prepared PURE_G4_-siRNA dendriplexes [90]. In this work, the goal was to develop a tool for gene silencing. The mechanism proposed for siRNA releasing was based on a ‘‘proton sponge’’ mechanism that allowed the dendriplex to escape from the endosome and deliver siRNA to the cell. 

Applied to gene therapy, PPI dendrimers were modified with maltose (mal-PPI) and conjugated to single-chain fragment variables (scFvs). This system works as a carrier for siRNAs and improves siRNA delivery in EGFRvIII-positive tumors [91]. 

**Figure 3:**
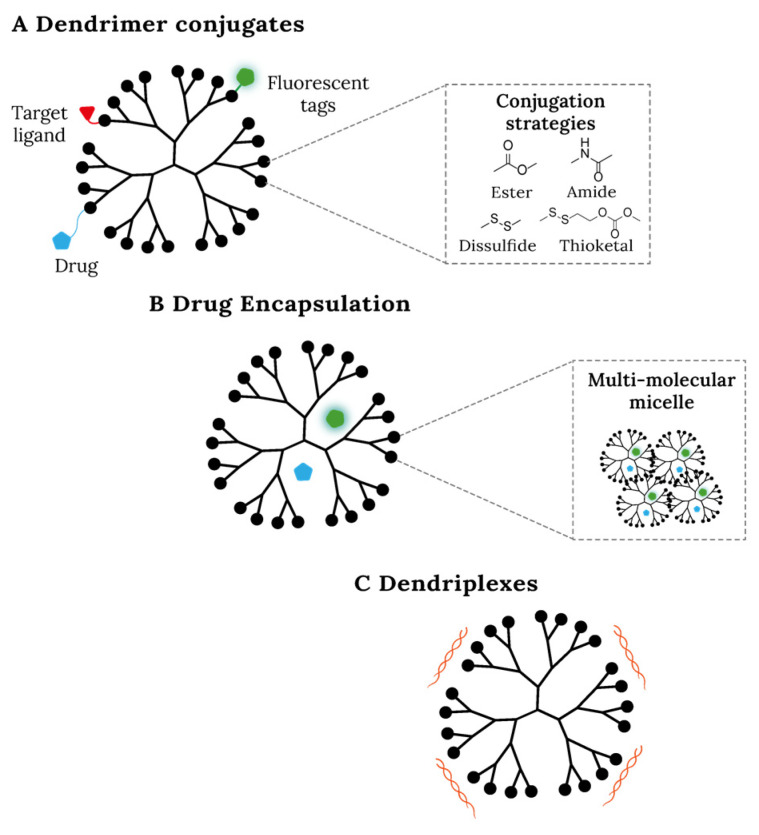
Dendrimer-based drug delivery systems. Different therapeutic strategies use dendrimers as nanocarriers. (**A**) Many ligands, drugs or fluorescent tags may be conjugated with dendrimers. Ester and amide groups are inserted in the surface to allow conjugation. When in the cell, these linkages are both cleaved by hydrolysis or enzymes. Another way is to directly conjugate dendrimers with specific groups such disulfide or thioketal linkers, which are cleaved by glutathione or reactive oxygen species (ROS). (**B**) Drugs and/or diagnostic agents are encapsulated into the hydrophobic pocket of dendrimers, a more efficient process in the case of hydrophobic compounds. The dendritic box or unimolecular micelle is promoted by guest–host interactions. Multi-molecular micelles are formed when conjugated dendrimers form agglomerates. (**C**) This is another way to use dendrimers as delivery systems, taking advantage of electrostatic interactions. Nucleic acids interact with the cationic surface groups (e.g, protonated amines) of the dendrimers.

The use of dendrimers and polymeric nanoparticle–aptamer bioconjugates can also be very useful for the development of effective delivery systems [92,93]. In this sense, it was found that PEGylated PAMAMs, functionalized with the anti-PSMA aptamer and loaded with the tumor suppressors non-coding miR-15a and miR-16-1, induce cell death of prostate cancer LNCaP and PC3 cell models [94]. Figure 3 and Table 4 summarizes the different strategies to use dendrimers as nanovesicles for drug and gene delivery. 

### 3.4. Dendrimers as Intrinsic Anticancer Drugs 

As highlighted in the previous sections, dendrimers have a remarkable capacity to act as therapeutics nanocarriers. Interestingly, a few studies extended the conventional role of these NPs to a new paradigm: the investigation of these macromolecules as anticancer agents (Table 5), taking advantage of intrinsic properties introduced by rational design. 

Xiao Zhang et al. evaluated the therapeutic potential of tryptophane-rich peptide dendrimers (TRPDs) against various tumor cell lines [95]. The results show that TRPDs display a high cytotoxicity towards various tumor cell lines and inhibited the proliferation of tumor cells using a BALB/c mice in vivo model. Another work demonstrated that two different CPP44 peptide–dendrimer conjugates were efficient against acute leukemia [96]. The co-administration of peptide dendrimers, composed of lysine and arginine residues, was shown to induce a significant enhancement of DOX and gemcitabine chemotherapeutic action in a mouse model of pancreatic ductal adenocarcinoma [97]. This work paved the way for the use of dendrimers against tumors with hypovascular and hypopermeable characteristics, including pancreatic ductal adenocarcinoma (a highly lethal and therapeutically resistant cancer).

Other dendrimer classes such as phosphorus and carbosilane dendrimers were shown to potentially act as anticancer agents [24,25]. These dendrimers were tested against several in vitro cancer cell models such as the solid tumor KB cell line, liquid tumor HL-60 cell line (phosphorus dendrimers), and the HeLa, HT-29, MCF-7, and MDA-MB-231 cell lines (carbosilane dendrimers).

Ornithine dendrimers also demonstrated good potential as anticancer drugs. His- and Pro-rich amphiphilic bola-type dendrimers were studied against U87 and T98G glioblastoma cells. Both ornithine-type dendrimers were efficient against both cell lines, showing a cytostatic effect [98]. 

In addition, Shao et al. demonstrated that dendrimers possess both intrinsic anticancer and antimetastatic properties [10]. In their study, a PEGylated polyacylthiourea (PATU_G4_-PEG) dendrimer was investigated. Upon performing tumor growth inhibition studies, these dendrimers, without conventional therapeutic agents in their composition, exhibited potent anticancer activity. One of the major advantages of PATU_G4_-PEG is the fact that it presents a low acute and subacute in vivo toxicity, since the major organs of the tested mice did not show significant damages or lesions after treatment and since, after five consecutive days post-treatment, there were no substantial changes in mouse serum chemistry. Using Doxil^®^, an established first-line anticancer PEGylated liposome formulation as the positive control, the authors demonstrated that PATU_G4_-PEG had a higher anticancer activity in the animal model. As a possible mechanism of action, they proposed that these NPs can sequestrate copper, allowing the inhibition of angiogenesis and cellular proliferation. The authors also reported the inhibition of cell seeding and consequently a strong decrease in cell metastasis by comparing with mice treated with PBS as a control group and mice treated with ammonium tetrathiomolybdate (TM), a strong copper-depleting agent for cancer treatment under clinical trials [10]. Although further studies are needed to validate the mechanisms of action, this study hypothesized a central role of dendrimers in cancer therapies, changing the way we look at dendrimers and opening new trends for future research.

## 4. Dendrimers Cellular Uptake and Mechanism of Action at Cell Organelle Level

**Figure 4:**
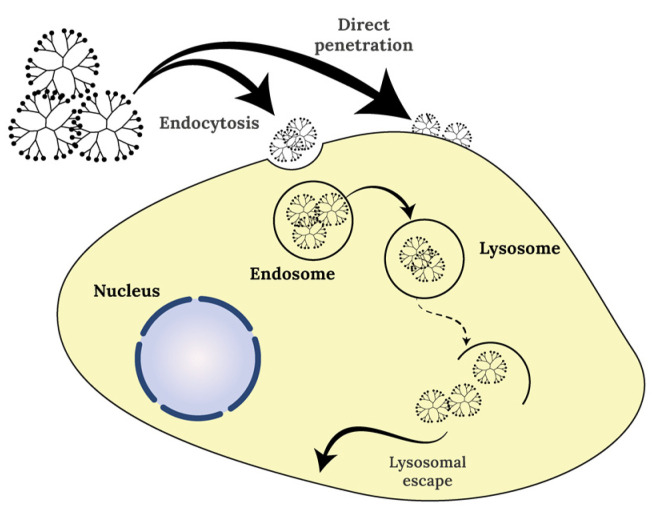
Schematic representation of dendrimer intracellular transportation. In general, dendrimers enter the target cells via direct penetration or endocytosis pathways. At cell level, dendrimers that follow the endocytic pathway are released from endosomes and migrate to lysosomes. Then these macromolecules can be released from lysosomes through a ‘’proton-sponge’’ effect as shown for poly(propylene imine) dendrimers. Adapted from [79].

Dendrimers and other nanosized particles naturally accumulate in tumor sites by a possible enhanced permeability and retention (EPR) effect. This happens because the tumor microenvironment is rich in blood vessels due to increased angiogenesis. Using the blood vessels surrounding tumors, the nanoparticles can be driven into the tumors, crossing cellular barriers by transcellular or paracellular pathways [34,79]. The main question at this point is: how does the intracellular transportation of these macromolecules occur? In the case of dendrimers, their entry into target cells via direct penetration or endocytosis pathways has been described [99] (Figure 4). Importantly, the biodistribution and retention of the nanoparticles are the foremost determinants, since their diffusion and adhesion properties significantly depend on size and uptake efficiencies [100]. NPs possess biomimetic features due to their size, which is in the same range of biomolecules such as antibodies, nucleic acids, proteins, and membrane receptors. Cellular uptake, toxicity, targeting, and intracellular trafficking of NPs can be optimized by tuning physicochemical properties of NP such as size, shape, and surface properties [101]. These properties determine their uptake into mammalian cells, normally achieved by endocytosis, a form of active transport that can be classified into two major categories: phagocytosis and pinocytosis [101].

Briefly, phagocytosis occurs in specialized cells, designated phagocytes, and consists of the internalization of large particles such as debris, bacteria or other large-size solutes. In this process, the target particle is coated with specific molecules (opsonins) that trigger its internalization and is then ingested by the cell and compartmentalized to a phagosome (plasma-membrane-derived vesicle). In the intracellular space, the phagosome fuses with the lysosome and the particle is digested at acidic pH. On the other hand, pinocytosis is a continuous process that consists in the formation of a plasma membrane invagination to capture small droplets of extracellular fluid and the molecules dissolved in it, which can be, for instance, biomolecules and nutrients. Pinocytosis can be subcategorized into clathrin-mediated endocytosis, caveolae-mediated endocytosis, and macropinocytosis, depending on the aim and specificity of the process.

Kitchens et al. studied the cellular uptake of dendrimers. Their work demonstrated that cationic and anionic PAMAM dendrimers enter the cells by a endocytosis mechanism [102]. Using lysosomal marker protein 1 (LAMP1), the authors observed that PAMAM_G2_-NH_2_ and PAMAM_G1.5_-CO_2_H dendrimers co-localized at the lysosome level at different time points depending on dendrimer surface charge. The authors also observed a decrease in PAMAM_G4_-NH_2_ uptake when the endocytic pathway was blocked using different inhibitors (brefeldin A, colchicine, filipin) [103]. In HeLa cells (cervical cancer cells), PAMAM dendrimers were shown to be internalized by two major mechanisms: clathrin-dependent endocytosis and macropinocytosis [104]. 

In addition to endocytosis, the uptake of polycationic polymers by cancer cells can follow a direct penetration pathway (with or without pore formation). Seungpyo Hong et al. reported the interaction of cationic dendrimers with supported lipid bilayers. The main conclusion was that these NPs can enter a cell membrane model by inducing pore formation. Applying atomic force microscopy (AFM), the authors observed that cationic PAMAM dendrimers can form holes/pores in the lipidic membrane and can also remove lipidic molecules from existing membrane defects. The authors also evaluated cell membrane recovery after dendrimer removal and found that hole/pore formation can be reversible [105]. The pore-forming ability of cationic molecules such as dendrimers and peptides can be used to develop formulations for anticancer therapy, as this mechanism of action can potentially lead to cell death [106,107].

Importantly, positively charged surface groups produce a localized charge density that is known to influence the interaction (and consequent toxicity) of dendrimers with cell membranes that possess a relevant content of negatively charged groups (as observed for the plasma membrane of cancer cells) [49]. Cellular permeability is also affected by the dendrimer generation (i.e., size). For instance, PAMAM_G2_ shows higher permeability rates than PAMAM_G4_ [108]. 

**Figure 5:**
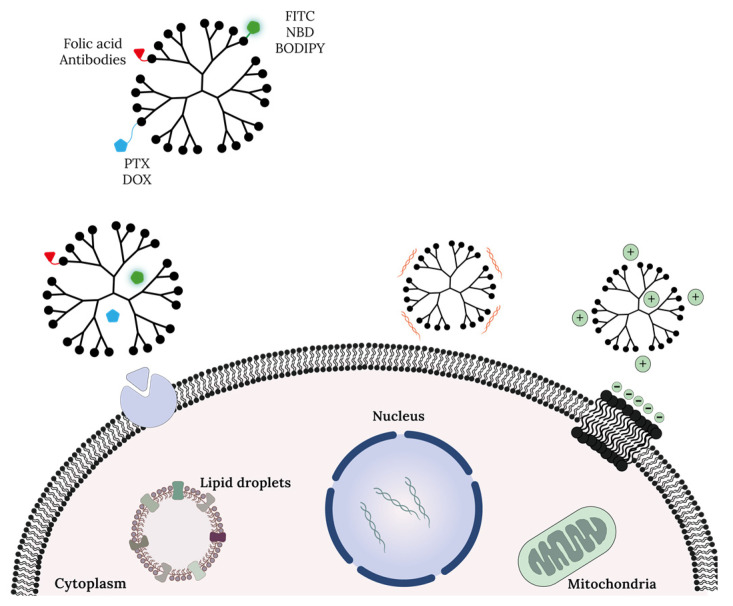
Schematic representation of potential intracellular cellular organelles (including plasma membrane, lipid droplets, cell nucleus, and mitochondria) targeted by dendrimers used in anticancer strategies. Cationic surface of cationic modified dendrimers is known to strongly interact with negatively charged lipids present in cancer cell membranes. Cationic dendrimers are also shown to specifically target mitochondria.

Dendrimers can then be designed to target a specific organelle or tissue (with a distinct mechanism of action) and induce, for instance, less side effects to normal/healthy cells. Chemical modifications in the dendrimer core or surface can improve dendrimer–cell interactions, as illustrated bellow (Figure 5). Aleksandra Szwed et al. studied the interaction mechanisms of hybrid carbosilane–viologen–phosphorus dendrimers (SMT1 and SMT2) with two different murine cell lines. These dendrimers present two distinct cationic groups (internal and outer) that are specific for mitochondria, inducing perturbations in mitochondrial membrane potential and the formation of reactive oxigen species (ROS) [109]. 

In another study, the impact and target of PAMAM dendrimer generation (G4, G5, G6) on mitochondria and other cellular organelles such as lysosomes was evaluated in HaCaT (human epidermal keratinocyte cells) and SW480 (primary adenocarcinoma) cell lines [63]. In general, SW4810 cells were more sensitive to these cationic dendrimers than HaCaT cells. After treatment, the production of ROS was higher in SW480 and reached a maximum after 4 h of exposure. These results suggest the specificity of PAMAM dendrimers towards cancer cell mitochondria in comparison to normal cells [110]. The increase in ROS and the expression of apoptotic markers such as BAX and PARP are well reported for the activity of dendrimers used in anticancer strategies [111].

The therapeutic potential of TRPDs in several in vitro models (HepG2, MCF-7 or SKOV3 cells) was evaluated as described above [95]. The results show a good cytotoxic profile against chemoresistance cell lines (MCF-7/ADR and SKOV3/ADR cells), and it was found that the mechanism of action is related to significant supramolecular interactions with DNA through the tryptophan residues [95].

One of the main patterns of cancer cells is the dysregulation of lipid metabolism, leading to a high content of lipid droplets (LDs). LDs are subcellular organelles with nano to micron diameter sizes with the ability to store high amounts of cholesterol or fatty acids, thus allowing cancer cells to avoid cytotoxic processes. The high fatty acid content can also provide an extra source of energy to cancer cells. Importantly, LDs are also associated with proliferation, invasion, metastasis, and chemoresistance processes. Thus, LDs can be viewed as future cancer hallmarks. Intracellular targeting of lipid droplets is also now becoming an imaging tool to monitor cancer cells and the targets of innovative therapeutic approaches [112]. Using “click” chemistry, multivalent niacin–polymeric (including dendrimer) conjugates were shown to target LDs [113]. The production of nitric oxide (NO) following treatment was significantly increased and a reduction in a LD relevant enzyme (diacylglycerol acyltransferase, DGAT2) involved in triglyceride biosynthesis was observed [112]. This strategy can be applied in the future for the treatment of cancer and other diseases that involve the accumulation of triglycerides/LDs.

## 5. Conclusions

In the last decades, many cases of toxicity associated with distinct chemotherapeutics have been reported, such as the case of paclitaxel (PTX), a microtubule stabilizing agent commonly used to treat breast and ovarian cancer. High doses of PTX were found to induce neutropenia, i.e., low concentration of neutrophils, neuropathy, and loss of hair in patients with metastatic breast cancer. It has also been demonstrated that the anticancer agent doxorubicin (DOX) can promote ovarian toxicity effects in cancer patients. In addition to high toxicity levels, one of the major problems associated with conventional chemotherapy is drug resistance, either because the initial tumor fails to respond to the treatment or because it acquires resistance during relapse. Therefore, chemoresistance is a critical limitation to the application of chemotherapy and it must be considered when designing new therapeutics.

To surpass these shortcomings, several types of dendrimers and other nanocarriers have been already reported and are revolutionizing the field of nanomedicine. In this regard, dendrimers were found to be extraordinary nanocarriers for drug and nucleic acids, and their use allowed an increase in therapeutic efficacy. Their use as nanocarriers has many advantages including increased drug solubilization and drug bioavailability and allowing for nucleic acid transportation. In addition, the fact that dendrimers (at least PAMAM dendrimers) can cross the blood–brain barrier makes them extremely appealing for a variety of related therapeutics, such as the treatment of glioblastomas. 

In the last decade, dendrimers have started to be explored as intrinsic anticancer drugs. For that, several groups used cationic dendrimers as mimics of cationic anticancer peptides. This strategy takes advantage of the enhanced electrostatic interactions between cationic dendrimers and cancer cells. This is due to the cancer cells having negatively charged surfaces arising from lactate overproduction and altered membrane glycosylation patterns. In contrast, healthy mammalian cell membranes are largely zwitterionic. Importantly, anticancer dendrimers demonstrated the capacity to strongly decrease cell metastasis.

In a different but complementary scenario, dendrimers are also powerful tools for the bioimaging of cancer cells, helping medical diagnosis. 

Despite all their potential in cancer treatment, dendrimers can display significant cytotoxicity and trigger hemolysis. In most cases, toxicity is related to the strong cationic characteristics associated with amine-terminated dendrimers, and this was found to be size/charge/*M*_w_ dependent. High charge and strong interaction with negatively charged cell surfaces can cause destabilization and lysis. Nonetheless, PEGylation has been shown to reduce toxicity and improve pharmacokinetics. Additionally, dendrimer modifications with targeting agents, such as tumor-specific antibodies or folic acid, decrease the number of reactive groups on the surface of dendrimers, thus reducing their toxicity and increasing their specificity. 

In summary, dendrimers are now foreseen as outstanding chemotherapeutics, either via nanoformulation (drug nanocarriers, drug nanoconjugates, dendriplexes) or as polymer drugs (innate anticancer and anti-metastatic agents). This versatility is certainly a key driving force for their translation to clinics in the near future.

## Figures and Tables

**Table 1:** Summary of different classes of dendrimers depicted in above section.

Classes of Dendrimers	Chemical Structure
PAMAM	Ethylenediamine-based core and terminal groups with primary amines
PPL	Amino acid lysine-base core and branching units
PPI	1,4-Diaminobutane-based core and terminal groups with primary amines
Phosphorous dendrimers	P-Cl-based core, azabisphosphonates are possible terminal groups
Carbosilane dendrimers	Si-based dendrimer

**Table 2:** Suitable properties of dendrimers to be used as delivery systems in different strategies.

Properties	Observations	References
Low polydispersity index	Common to all classes of dendrimers	[37,38]
EPR effect	Size/generation/*M*_w_ dependent	[39]
Permeability towards BBB	Already observed for PAMAM dendrimers	[40,41,42,43]
Highly solubility	Common to the majority of dendrimer classes	[44,45]
Multifunctional platform	Common to all classes of dendrimers	[46]
Highly loading capacity	Size/generation/*M*_w_ dependent	[47]
Stability	Common to all classes of dendrimers	[39]
Low toxicity and immunogenicity	Size/generation/*M*_w_/charge dependent	[48,49,50]

EPR: enhanced permeability and retention, BBB: blood–brain barrier.

**Table 4:** Summary of selected dendrimers used as drug and gene nanocarriers.

Nanoformulation	Therapeutic	In Vitro Cell Cancer Model	Reference
DOX-PAMAM	Doxorubicin	Metastatic lung cancer (B16-F10 melanoma cells)	[73]
Btz loaded PAMAM	Bortezomib	Breast cancer (MDA-MB-231 cells)	[75]
YIGSR-CMCht/PAMAM	Gemcitabine	Colorectal cancer (HCT-116 cells)	[76]
DOX-Tz-PAMAM	Doxorubocin	Breast cancer (SKBR-3 and MCF-7 cells)	[77]
PTX-biotinylated-PAMAM	Paclitaxel	Ovarian cancer (OVCAR-3 cells)	[78]
FA-FITC-MTX-PAMAM	Methotrexate	Human epithelial carcinoma (KB cells)	[5]
MTX-FA-PPI	Methotrexate	Breast cancer (MCF-7 cells)	[80]
mAbk1-PPI-PTX	Paclitaxel	Ovarian cancer (OVCAR-3 cells)	[81]
5-FU-lysine dendrimers	5′-Fluoroacil	Lung cancer (A549 cells), cervical cancer (HeLa cells), and hepatocellular carcinoma (HepG2 cells)	[83]
PTX@PURE-OMeOx_48_ PTX@PURE-OEtOx_48_	Paclitaxel	Hepatocellular carcinoma	[84]
PAMAM-FA/siVEGFA	siVEGFA (vascular endothelial growth factor)	Head and neck squamous cell carcinoma (HN12 cells)	[88]
DOX-PAMAM-HA/MVP-siRNA	Doxorubin and siRNA Major Vault Protein	Breast cancer (MCF-7/ADR cells)	[89]
mal-PPI/siRNA	siRNA Epidermal growth factor variant III	EGFRvIII positive tumors	[91]
PEGylated PAMAM	Non-coding miR-15a and miR-16-1	Prostate cancer cells (LNCaP and PC3)	[94]

**Table 5:** Selected dendrimers used as anticancer drugs.

Dendrimers	In Vitro Cell Cancer Model	Reference
TRPDs	Ovarian cancer (SKOV3 and SKOV3/ADR), breast cancer (MCF-7 and MCF7/ADR cells)	[95]
CPP4 peptide-PAMAM conjugates	Acute myeloid leukemia (AML3639 and AML0934 cells)	[96]
Lysine and arginine dendrimers	Pancreatic ductal carcinoma	[97]
Phosphorus dendrimers	Solid tumor (KB cells), Liquid tumor (HL-60 cells)	[24]
Carbosilane dendrimers	Cervical cancer (HeLa cells), Colon cancer (HT-29 cells), Breast cancer (MCF-7 and MDA-MB-231 cells),	[25]
Ornithine dendrimers	Glioblastoma (U87 cells)	[98]
PATU_G4_-PEG	Breast cancer (MCF-7/ADR cells)	[10]

## Data Availability

Not applicable.

## References

[1] Hassanpour S.H., Dehghani M. (2017). Review of cancer from perspective of molecular. J. Cancer Res. Pract..

[2] McGuire S. (2016). World Cancer Report 2014. Geneva, Switzerland: World Health Organization, International Agency for Research on Cancer, WHO Press, 2015. Adv. Nutr..

[3] Parsa N. (2012). Environmental factors inducing human cancers. Iran J. Public Health.

[4] Pérez-Herrero E., Fernández-Medarde A. (2015). Advanced targeted therapies in cancer: Drug nanocarriers, the future of chemotherapy. Eur. J. Pharm. Biopharm.

[5] Quintana A., Raczka E., Piehler L., Lee I., Myc A., Majoros I., Patri A.K., Thomas T., Mulé J., Baker J.R. (2002). Design and function of a dendrimer-based therapeutic nanodevice targeted to tumor cells through the folate receptor. Pharm. Res..

[6] Wiener E.C., Brechbiel M.W., Brothers H., Magin R.L., Gansow O.A., Tomalia D.A., Lauterbur P.C. (1994). Dendrimer-based metal chelates: A new class of magnetic resonance imaging contrast agents. Magn. Reson. Med..

[7] Alibolandi M., Hoseini F., Mohammadi M., Ramezani P., Einafshar E., Taghdisi S.M., Ramezani M., Abnous K. (2018). Curcumin-entrapped MUC-1 aptamer targeted dendrimer-gold hybrid nanostructure as a theranostic system for colon adenocarcinoma. Int. J. Pharm..

[8] Restani R.B., Morgado P.I., Ribeiro M.P., Correia I.J., Aguiar-Ricardo A., Bonifácio V.D. (2012). Biocompatible polyurea dendrimers with pH-dependent fluorescence. Angew. Chem. Int. Ed. Engl..

[9] Wu Y., Sefah K., Liu H., Wang R., Tan W. (2010). DNA aptamer-micelle as an efficient detection/delivery vehicle toward cancer cells. Proc. Natl. Acad. Sci. USA.

[10] Shao S., Zhou Q., Si J., Tang J., Liu X., Wang M., Gao J., Wang K., Xu R., Shen Y. (2017). A non-cytotoxic dendrimer with innate and potent anticancer and anti-metastatic activities. Nat. Biomed. Eng..

[11] Sanvicens N., Marco M.P. (2008). Multifunctional nanoparticles--properties and prospects for their use in human medicine. Trends Biotechnol..

[12] Rodríguez-Acosta G.L., Hernández-Montalbán C., Vega-Razo M.F.S., Castillo-Rodríguez I.O., Martínez-García M. (2022). Polymer-dendrimer Hybrids as Carriers of Anticancer Agents. Curr. Drug Targets.

[13] Buhleier E., Wehner W., VÖGtle F. (1978). “Cascade”- and “Nonskid-Chain-like” Syntheses of Molecular Cavity Topologies. Synthesis.

[14] Heegaard P.M., Boas U., Sorensen N.S. (2010). Dendrimers for vaccine and immunostimulatory uses. A review. Bioconjug Chem..

[15] Tomalia D.A., Baker H., Dewald J., Hall M., Kallos G., Martin S., Roeck J., Ryder J., Smith P. (1985). A New Class of Polymers: Starburst-Dendritic Macromolecules. Polym. J..

[16] Wu L.P., Ficker M., Christensen J.B., Trohopoulos P.N., Moghimi S.M. (2015). Dendrimers in Medicine: Therapeutic Concepts and Pharmaceutical Challenges. Bioconjug Chem..

[17] Hawker C.J., Frechet J.M.J. (1990). Preparation of polymers with controlled molecular architecture. A new convergent approach to dendritic macromolecules. J. Am. Chem. Soc..

[18] Kolb H.C., Finn M.G., Sharpless K.B. (2001). Click Chemistry: Diverse Chemical Function from a Few Good Reactions. Angew. Chem. Int. Ed. Engl..

[19] Dockery L., Daniel M.C. (2018). Dendronized Systems for the Delivery of Chemotherapeutics. Adv. Cancer Res..

[20] She W., Li N., Luo K., Guo C., Wang G., Geng Y., Gu Z. (2013). Dendronized heparin-doxorubicin conjugate based nanoparticle as pH-responsive drug delivery system for cancer therapy. Biomaterials.

[21] Lo S.T., Kumar A., Hsieh J.T., Sun X. (2013). Dendrimer nanoscaffolds for potential theranostics of prostate cancer with a focus on radiochemistry. Mol. Pharm..

[22] Palmerston Mendes L., Pan J., Torchilin V.P. (2017). Dendrimers as Nanocarriers for Nucleic Acid and Drug Delivery in Cancer Therapy. Molecules.

[23] Rahimi A., Amjad-Iranagh S., Modarress H. (2016). Molecular dynamics simulation of coarse-grained poly(L-lysine) dendrimers. J. Mol. Model..

[24] Caminade A.M. (2020). Phosphorus Dendrimers as Nanotools against Cancers. Molecules.

[25] Maroto-Díaz M., Elie B.T., Gómez-Sal P., Pérez-Serrano J., Gómez R., Contel M., Javier de la Mata F. (2016). Synthesis and anticancer activity of carbosilane metallodendrimers based on arene ruthenium(ii) complexes. Dalton Trans..

[26] Lee C.C., MacKay J.A., Fréchet J.M.J., Szoka F.C. (2005). Designing dendrimers for biological applications. Nat. Biotechnol..

[27] Abbasi E., Aval S.F., Akbarzadeh A., Milani M., Nasrabadi H.T., Joo S.W., Hanifehpour Y., Nejati-Koshki K., Pashaei-Asl R. (2014). Dendrimers: Synthesis, applications, and properties. Nanoscale Res. Lett..

[28] Noriega-Luna B., Godínez L.A., Rodríguez F.J., Rodríguez A., Zaldívar-Lelo de Larrea G., Sosa-Ferreyra C.F., Mercado-Curiel R.F., Manríquez J., Bustos E. (2014). Applications of Dendrimers in Drug Delivery Agents, Diagnosis, Therapy, and Detection. J. Nanomater..

[29] Duncan R., Izzo L. (2005). Dendrimer biocompatibility and toxicity. Adv. Drug Deliv. Rev..

[30] Choudhary S., Gupta L., Rani S., Dave K., Gupta U. (2017). Impact of Dendrimers on Solubility of Hydrophobic Drug Molecules. Front. Pharm..

[31] Alven S., Aderibigbe B.A. (2020). The Therapeutic Efficacy of Dendrimer and Micelle Formulations for Breast Cancer Treatment. Pharmaceutics.

[32] Trembleau L., Simpson M., Cheyne R.W., Escofet I., Appleyard M.V.C.A.L., Murray K., Sharp S., Thompson A.M., Smith T.A.D. (2011). Development of 18F-fluorinatable dendrons and their application to cancer cell targeting. New J. Chem..

[33] Liu M., Fréchet J.M. (1999). Designing dendrimers for drug delivery. Pharm. Sci. Technol. Today.

[34] Menjoge A.R., Kannan R.M., Tomalia D.A. (2010). Dendrimer-based drug and imaging conjugates: Design considerations for nanomedical applications. Drug Discov. Today.

[35] Wang J., Li B., Qiu L., Qiao X., Yang H. (2022). Dendrimer-based drug delivery systems: History, challenges, and latest developments. J. Biol. Eng..

[36] Najlah M., Freeman S., Attwood D., D’Emanuele A. (2007). In vitro evaluation of dendrimer prodrugs for oral drug delivery. Int. J. Pharm..

[37] Kolhe P., Khandare J., Pillai O., Kannan S., Lieh-Lai M., Kannan R. (2004). Hyperbranched polymer-drug conjugates with high drug payload for enhanced cellular delivery. Pharm. Res..

[38] Patel V., Rajani C., Paul D., Borisa P., Rajpoot K., Youngren-Ortiz S.R., Tekade R.K., Tekade R.K. (2020). Chapter 8—Dendrimers as novel drug-delivery system and its applications. Drug Delivery Systems.

[39] Rai D.B., Gupta N., Pooja D., Kulhari H., Chauhan A., Kulhari H. (2020). Dendrimers for diagnostic applications. Pharmaceutical Applications of Dendrimers.

[40] Zhao J., Zhang B., Shen S., Chen J., Zhang Q., Jiang X., Pang Z. (2015). CREKA peptide-conjugated dendrimer nanoparticles for glioblastoma multiforme delivery. J. Colloid. Interface Sci..

[41] Sharma A., Porterfield J.E., Smith E., Sharma R., Kannan S., Kannan R.M. (2018). Effect of mannose targeting of hydroxyl PAMAM dendrimers on cellular and organ biodistribution in a neonatal brain injury model. J. Control. Release.

[42] Srinageshwar B., Peruzzaro S., Andrews M., Johnson K., Hietpas A., Clark B., McGuire C., Petersen E., Kippe J., Stewart A. (2017). PAMAM Dendrimers Cross the Blood-Brain Barrier When Administered through the Carotid Artery in C57BL/6J Mice. Int. J. Mol. Sci..

[43] Srinageshwar B., Dils A., Sturgis J., Wedster A., Kathirvelu B., Baiyasi S., Swanson D., Sharma A., Dunbar G.L., Rossignol J. (2019). Surface-Modified G4 PAMAM Dendrimers Cross the Blood–Brain Barrier Following Multiple Tail-Vein Injections in C57BL/6J Mice. ACS Chem. Neurosci..

[44] Yiyun C., Tongwen X. (2005). Dendrimers as potential drug carriers. Part I. Solubilization of non-steroidal anti-inflammatory drugs in the presence of polyamidoamine dendrimers. Eur. J. Med. Chem..

[45] Hawker C.J., Wooley K.L., Fréchet J.M.J. (1993). Unimolecular micelles and globular amphiphiles: Dendritic macromolecules as novel recyclable solubilization agents. J. Chem. Soc. Perkin Trans..

[46] Vaidya A., Jain S., Pathak K., Pathak D. (2018). Dendrimers: Nanosized Multifunctional Platform for Drug Delivery. Drug Deliv. Lett..

[47] Singh J., Jain K., Mehra N.K., Jain N.K. (2016). Dendrimers in anticancer drug delivery: Mechanism of interaction of drug and dendrimers. Artif. Cells Nanomed. Biotechnol..

[48] Jain K., Kesharwani P., Gupta U., Jain N.K. (2010). Dendrimer toxicity: Let’s meet the challenge. Int. J. Pharm..

[49] Pinto S.N., Mil-Homens D., Pires R.F., Alves M.M., Serafim G., Martinho N., Melo M., Fialho A.M., Bonifácio V.D.B. (2022). Core–shell polycationic polyurea pharmadendrimers: New-generation of sustainable broad-spectrum antibiotics and antifungals. Biomater. Sci..

[50] Pryor J.B., Harper B.J., Harper S.L. (2014). Comparative toxicological assessment of PAMAM and thiophosphoryl dendrimers using embryonic zebrafish. Int. J. Nanomed..

[51] Kunjachan S., Jayapaul J., Mertens M.E., Storm G., Kiessling F., Lammers T. (2012). Theranostic systems and strategies for monitoring nanomedicine-mediated drug targeting. Curr. Pharm. Biotechnol..

[52] Ehling J., Lammers T., Kiessling F. (2013). Non-invasive imaging for studying anti-angiogenic therapy effects. Thromb. Haemost..

[53] Lanza G.M., Caruthers S.D., Winter P.M., Hughes M.S., Schmieder A.H., Hu G., Wickline S.A. (2010). Angiogenesis imaging with vascular-constrained particles: The why and how. Eur. J. Nucl. Med. Mol. Imaging.

[54] Misselwitz B., Schmitt-Willich H., Ebert W., Frenzel T., Weinmann H.J. (2001). Pharmacokinetics of Gadomer-17, a new dendritic magnetic resonance contrast agent. Magma.

[55] Jeelani S., Reddy R.C., Maheswaran T., Asokan G.S., Dany A., Anand B. (2014). Theranostics: A treasured tailor for tomorrow. J. Pharm. Bioallied Sci..

[56] Jo S.D., Ku S.H., Won Y.Y., Kim S.H., Kwon I.C. (2016). Targeted Nanotheranostics for Future Personalized Medicine: Recent Progress in Cancer Therapy. Theranostics.

[57] Rizzo L.Y., Theek B., Storm G., Kiessling F., Lammers T. (2013). Recent progress in nanomedicine: Therapeutic, diagnostic and theranostic applications. Curr. Opin. Biotechnol.

[58] Xu R., Wang Y., Wang X., Jeong E.K., Parker D.L., Lu Z.R. (2007). In Vivo evaluation of a PAMAM-cystamine-(Gd-DO3A) conjugate as a biodegradable macromolecular MRI contrast agent. Exp. Biol. Med..

[59] Kong L., Xing L., Zhou B., Du L., Shi X. (2017). Dendrimer-Modified MoS(2) Nanoflakes as a Platform for Combinational Gene Silencing and Photothermal Therapy of Tumors. ACS Appl. Mater. Interfaces.

[60] Zhang Q., Wang L., Jiang Y., Gao W., Wang Y., Yang X., Yang X., Liu Z. (2017). Gold Nanorods with Silica Shell and PAMAM Dendrimers for Efficient Photothermal Therapy and Low Toxic Codelivery of Anticancer Drug and siRNA. Adv. Mater. Interfaces.

[61] Ghai A., Singh B., Panwar Hazari P., Schultz M.K., Parmar A., Kumar P., Sharma S., Dhawan D., Kumar Mishra A. (2015). Radiolabeling optimization and characterization of (68)Ga labeled DOTA-polyamido-amine dendrimer conjugate—Animal biodistribution and PET imaging results. Appl. Radiat. Isot..

[62] Ma W., Fu F., Zhu J., Huang R., Zhu Y., Liu Z., Wang J., Conti P.S., Shi X., Chen K. (2018). 64Cu-Labeled multifunctional dendrimers for targeted tumor PET imaging. Nanoscale.

[63] Albertazzi L., Storti B., Marchetti L., Beltram F. (2010). Delivery and subcellular targeting of dendrimer-based fluorescent pH sensors in living cells. J. Am. Chem. Soc..

[64] Saovapakhiran A., D’Emanuele A., Attwood D., Penny J. (2009). Surface modification of PAMAM dendrimers modulates the mechanism of cellular internalization. Bioconjug Chem..

[65] Xiao T., Li D., Shi X., Shen M. (2020). PAMAM Dendrimer-Based Nanodevices for Nuclear Medicine Applications. Macromol. Biosci..

[66] Lesniak W.G., Mishra M.K., Jyoti A., Balakrishnan B., Zhang F., Nance E., Romero R., Kannan S., Kannan R.M. (2013). Biodistribution of Fluorescently Labeled PAMAM Dendrimers in Neonatal Rabbits: Effect of Neuroinflammation. Mol. Pharm..

[67] Kobayashi H., Kawamoto S., Saga T., Sato N., Hiraga A., Konishi J., Togashi K., Brechbiel M.W. (2001). Micro-MR angiography of normal and intratumoral vessels in mice using dedicated intravascular MR contrast agents with high generation of polyamidoamine dendrimer core: Reference to pharmacokinetic properties of dendrimer-based MR contrast agents. J. Magn. Reson. Imaging.

[68] Boyd B.J., Kaminskas L.M., Karellas P., Krippner G., Lessene R., Porter C.J.H. (2006). Cationic Poly-l-lysine Dendrimers:  Pharmacokinetics, Biodistribution, and Evidence for Metabolism and Bioresorption after Intravenous Administration to Rats. Mol. Pharm..

[69] Bhadra D., Bhadra S., Jain N.K. (2005). PEGylated peptide-based dendritic nanoparticulate systems for delivery of artemether. J. Drug Deliv. Sci. Technol..

[70] Kobayashi H., Saga T., Kawamoto S., Sato N., Hiraga A., Ishimori T., Konishi J., Togashi K., Brechbiel M.W. (2001). Dynamic micro-magnetic resonance imaging of liver micrometastasis in mice with a novel liver macromolecular magnetic resonance contrast agent DAB-Am64-(1B4M-Gd)(64). Cancer Res..

[71] Chis A.A., Dobrea C., Morgovan C., Arseniu A.M., Rus L.L., Butuca A., Juncan A.M., Totan M., Vonica-Tincu A.L., Cormos G. (2020). Applications and Limitations of Dendrimers in Biomedicine. Molecules.

[72] Bober Z., Bartusik-Aebisher D., Aebisher D. (2022). Application of Dendrimers in Anticancer Diagnostics and Therapy. Molecules.

[73] Zhong Q., Bielski E.R., Rodrigues L.S., Brown M.R., Reineke J.J., da Rocha S.R. (2016). Conjugation to Poly(amidoamine) Dendrimers and Pulmonary Delivery Reduce Cardiac Accumulation and Enhance Antitumor Activity of Doxorubicin in Lung Metastasis. Mol. Pharm..

[74] Wang H., Huang Q., Chang H., Xiao J., Cheng Y. (2016). Stimuli-responsive dendrimers in drug delivery. Biomater. Sci..

[75] Wang M., Wang Y., Hu K., Shao N., Cheng Y. (2015). Tumor extracellular acidity activated “off-on” release of bortezomib from a biocompatible dendrimer. Biomater Sci..

[76] Carvalho M.R., Carvalho C.R., Maia F.R., Caballero D., Kundu S.C., Reis R.L., Oliveira J.M. (2019). Peptide-Modified Dendrimer Nanoparticles for Targeted Therapy of Colorectal Cancer. Adv. Ther..

[77] Marcinkowska M., Sobierajska E., Stanczyk M., Janaszewska A., Chworos A., Klajnert-Maculewicz B. (2018). Conjugate of PAMAM Dendrimer, Doxorubicin and Monoclonal Antibody-Trastuzumab: The New Approach of a Well-Known Strategy. Polymers.

[78] Yao H., Ma J. (2018). Dendrimer-paclitaxel complexes for efficient treatment in ovarian cancer: Study on OVCAR-3 and HEK293T cells. Acta Biochim. Pol..

[79] Singh V., Sahebkar A., Kesharwani P. (2021). Poly (propylene imine) dendrimer as an emerging polymeric nanocarrier for anticancer drug and gene delivery. Eur. Polym. J..

[80] Kaur A., Jain K., Mehra N.K., Jain N.K. (2017). Development and characterization of surface engineered PPI dendrimers for targeted drug delivery. Artif. Cells Nanomed. Biotechnol..

[81] Jain N.K., Tare M.S., Mishra V., Tripathi P.K. (2015). The development, characterization and in vivo anti-ovarian cancer activity of poly(propylene imine) (PPI)-antibody conjugates containing encapsulated paclitaxel. Nanomedicine.

[82] Singh A., Trivedi P., Jain N.K. (2018). Advances in siRNA delivery in cancer therapy. Artif. Cells Nanomed Biotechnol..

[83] Zhao J., Zhou R., Fu X., Ren W., Ma L., Li R., Zhao Y., Guo L. (2014). Cell-penetrable lysine dendrimers for anti-cancer drug delivery: Synthesis and preliminary biological evaluation. Arch. Pharm..

[84] Restani R.B., Conde J., Pires R.F., Martins P., Fernandes A.R., Baptista P.V., Bonifácio V.D.B., Aguiar-Ricardo A. (2015). POxylated Polyurea Dendrimers: Smart Core-Shell Vectors with IC50 Lowering Capacity. Macromol. Biosci..

[85] Restani R.B., Pires R.F., Tolmatcheva A., Cabral R., Baptista P.V., Fernandes A.R., Casimiro T., Bonifácio V.D.B., Aguiar-Ricardo A. (2018). POxylated Dendrimer-Based Nano-in-Micro Dry Powder Formulations for Inhalation Chemotherapy. ChemistryOpen.

[86] Sridharan K., Gogtay N.J. (2016). Therapeutic nucleic acids: Current clinical status. Br. J. Clin. Pharm..

[87] Sonawane N.D., Szoka F.C., Verkman A.S. (2003). Chloride accumulation and swelling in endosomes enhances DNA transfer by polyamine-DNA polyplexes. J. Biol. Chem..

[88] Xu L., Yeudall W.A., Yang H. (2017). Folic acid-decorated polyamidoamine dendrimer exhibits high tumor uptake and sustained highly localized retention in solid tumors: Its utility for local siRNA delivery. Acta Biomater..

[89] Han M., Lv Q., Tang X.J., Hu Y.L., Xu D.H., Li F.Z., Liang W.Q., Gao J.Q. (2012). Overcoming drug resistance of MCF-7/ADR cells by altering intracellular distribution of doxorubicin via MVP knockdown with a novel siRNA polyamidoamine-hyaluronic acid complex. J. Control. Release.

[90] Restani R.B., Conde J., Baptista P.V., Cidade M.T., Bragança A.M., Morgado J., Correia I.J., Aguiar-Ricardo A., Bonifácio V.D.B. (2014). Polyurea dendrimer for efficient cytosolic siRNA delivery. RSC Adv..

[91] Tietze S., Schau I., Michen S., Ennen F., Janke A., Schackert G., Aigner A., Appelhans D., Temme A. (2017). A Poly(Propyleneimine) Dendrimer-Based Polyplex-System for Single-Chain Antibody-Mediated Targeted Delivery and Cellular Uptake of SiRNA. Small.

[92] Mignani S., Shi X., Ceña V., Majoral J.-P. (2020). Dendrimer– and polymeric nanoparticle–aptamer bioconjugates as nonviral delivery systems: A new approach in medicine. Drug Discov. Today.

[93] Alshaer W., Hillaireau H., Fattal E. (2018). Aptamer-guided nanomedicines for anticancer drug delivery. Adv. Drug Deliv. Rev..

[94] Wu X., Ding B., Gao J., Wang H., Fan W., Wang X., Zhang W., Wang X., Ye L., Zhang M. (2011). Second-generation aptamer-conjugated PSMA-targeted delivery system for prostate cancer therapy. Int. J. Nanomed..

[95] Zhang X., Zhang Z., Xu X., Li Y., Li Y., Jian Y., Gu Z. (2015). Bioinspired therapeutic dendrimers as efficient peptide drugs based on supramolecular interactions for tumor inhibition. Angew. Chem. Int. Ed. Engl..

[96] Kojima C., Saito K., Kondo E. (2018). Design of peptide–dendrimer conjugates with tumor homing and antitumor effects. Res. Chem. Intermed..

[97] Huang S., Huang X., Yan H. (2022). Peptide dendrimers as potentiators of conventional chemotherapy in the treatment of pancreatic cancer in a mouse model. Eur. J. Pharm. Biopharm..

[98] Cieślak M., Ryszawy D., Pudełek M., Urbanowicz M., Morawiak M., Staszewska-Krajewska O., Czyż J., Urbańczyk-Lipkowska Z. (2020). Bioinspired Bola-Type Peptide Dendrimers Inhibit Proliferation and Invasiveness of Glioblastoma Cells in a Manner Dependent on Their Structure and Amphipathic Properties. Pharmaceutics.

[99] Zhang J., Liu D., Zhang M., Sun Y., Zhang X., Guan G., Zhao X., Qiao M., Chen D., Hu H. (2016). The cellular uptake mechanism, intracellular transportation, and exocytosis of polyamidoamine dendrimers in multidrug-resistant breast cancer cells. Int. J. Nanomed..

[100] Shinde Patil V.R., Campbell C.J., Yun Y.H., Slack S.M., Goetz D.J. (2001). Particle diameter influences adhesion under flow. Biophys. J..

[101] Bamrungsap S., Zhao Z., Chen T., Wang L., Li C., Fu T., Tan W. (2012). Nanotechnology in therapeutics: A focus on nanoparticles as a drug delivery system. Nanomedicine.

[102] Kitchens K.M., Foraker A.B., Kolhatkar R.B., Swaan P.W., Ghandehari H. (2007). Endocytosis and interaction of poly (amidoamine) dendrimers with Caco-2 cells. Pharm. Res..

[103] Kitchens K.M., Kolhatkar R.B., Swaan P.W., Ghandehari H. (2008). Endocytosis inhibitors prevent poly(amidoamine) dendrimer internalization and permeability across Caco-2 cells. Mol. Pharm..

[104] Albertazzi L., Serresi M., Albanese A., Beltram F. (2010). Dendrimer internalization and intracellular trafficking in living cells. Mol. Pharm..

[105] Hong S., Bielinska A.U., Mecke A., Keszler B., Beals J.L., Shi X., Balogh L., Orr B.G., Baker J.R., Banaszak Holl M.M. (2004). Interaction of Poly(amidoamine) Dendrimers with Supported Lipid Bilayers and Cells:  Hole Formation and the Relation to Transport. Bioconjugate Chem..

[106] Jahanafrooz Z., Mokhtarzadeh A. (2022). Pore-forming Peptides: A New Treatment Option for Cancer. Curr. Med. Chem..

[107] Chernyshova D.N., Tyulin A.A., Ostroumova O.S., Efimova S.S. (2022). Discovery of the Potentiator of the Pore-Forming Ability of Lantibiotic Nisin: Perspectives for Anticancer Therapy. Membranes.

[108] Tajarobi F., El-Sayed M., Rege B.D., Polli J.E., Ghandehari H. (2001). Transport of poly amidoamine dendrimers across Madin-Darby canine kidney cells. Int. J. Pharm..

[109] Szwed A., Miłowska K., Michlewska S., Moreno S., Shcharbin D., Gomez-Ramirez R., de la Mata F.J., Majoral J.P., Bryszewska M., Gabryelak T. (2020). Generation Dependent Effects and Entrance to Mitochondria of Hybrid Dendrimers on Normal and Cancer Neuronal Cells In Vitro. Biomolecules.

[110] Mukherjee S.P., Lyng F.M., Garcia A., Davoren M., Byrne H.J. (2010). Mechanistic studies of in vitro cytotoxicity of poly(amidoamine) dendrimers in mammalian cells. Toxicol. Appl. Pharm..

[111] Czarnomysy R., Muszyńska A., Rok J., Rzepka Z., Bielawski K. (2021). Mechanism of Anticancer Action of Novel Imidazole Platinum(II) Complex Conjugated with G2 PAMAM-OH Dendrimer in Breast Cancer Cells. Int. J. Mol. Sci..

[112] Antunes P., Cruz A., Barbosa J., Bonifácio V.D.B., Pinto S.N. (2022). Lipid Droplets in Cancer: From Composition and Role to Imaging and Therapeutics. Molecules.

[113] Sharma A., Khatchadourian A., Khanna K., Sharma R., Kakkar A., Maysinger D. (2011). Multivalent niacin nanoconjugates for delivery to cytoplasmic lipid droplets. Biomaterials.

